# Relationship of joint hypermobility with low Back pain and lumbar spine osteoarthritis

**DOI:** 10.1186/s12891-019-2523-2

**Published:** 2019-04-09

**Authors:** Adam P. Goode, Rebecca J. Cleveland, Todd A. Schwartz, Amanda E. Nelson, Virginia B. Kraus, Howard J. Hillstrom, Marian T. Hannan, Portia Flowers, Jordan B. Renner, Joanne M. Jordan, Yvonne M. Golightly

**Affiliations:** 1 Department of Orthopedic Surgery, Duke University School of Medicine, Durham, NC USA; 2 Duke Clinical Research Institute, Duke University, Durham, NC USA; 3 Duke Department of Population Health Sciences, Durham, NC USA; 4 Thurston Arthritis Research Center, University of North Carolina, Chapel Hill, NC USA; 5 Department of Medicine, University of North Carolina, Chapel Hill, NC USA; 6 Department of Biostatistics, University of North Carolina, Chapel Hill, NC USA; 7 Duke Molecular Physiology Institute and Department of Medicine, Duke University School of Medicine, Durham, NC USA; 8 Hospital for Special Surgery, New York, NY USA; 9 Institute for Aging Research, Hebrew Senior Life, and Harvard Medical School, Boston, MA USA; 10 Department of Radiology, University of North Carolina, Chapel Hill, NC USA; 11 Department of Orthopedics, University of North Carolina, Chapel Hill, NC USA; 12 Department of Epidemiology, University of North Carolina, Chapel Hill, NC USA; 13 Injury Prevention Research Center, University of North Carolina, Chapel Hill, NC USA

**Keywords:** Low back pain, Hypermobility, Osteoarthritis, Lumbar spine, Intervertebral disc, Facet joint

## Abstract

**Background:**

Chronic low back pain (cLBP) affects millions of Americans and costs billions. Studies suggest a link between cLBP and joint hypermobility.

**Methods:**

We conducted cross-sectional primary analyses of joint hypermobility and cLBP, lumbar spine osteoarthritis (OA), and lumbar facet joint OA (FOA) in 3 large studies—the Generalized Osteoarthritis Study, Genetics of Generalized Osteoarthritis Study, and Johnston County Osteoarthritis Project (total *n* = 5072). Associations of joint hypermobility and Beighton trunk flexion with cLBP and lumbar OA were estimated using separate adjusted logistic regression models. Adjusted pooled odds ratios (pORs) and 95% confidence intervals (CIs) were then summarized—using random effect univariate, multivariate crude, and adjusted models—and heterogeneity was determined (I^2^ statistic).

**Results:**

In univariate models, hypermobility was associated with symptomatic FOA (pOR = 0.64 [95% CI 0.44, 0.93]) but this result was not found in the multivariate models. In multivariate adjusted models, hypermobility was not significantly associated with cLBP and lumbar OA, but trunk flexion was inversely associated with cLBP (pOR = 0.40 [95% 0.26, 0.62]), spine OA (pOR = 0.66 [95% CI 0.50, 0.87]), symptomatic spine OA (pOR = 0.39 [95% CI 0.28, 0.53]), and symptomatic FOA (pOR = 0.53 [95% CI 0.37, 0.77]). Generally, between-study heterogeneity was moderate-high.

**Conclusions:**

Hypermobility was not associated with cLBP or lumbar OA. The inverse association of trunk flexion with cLBP and lumbar OA may indicate a role for a flexible spine in avoiding or managing these conditions.

**Electronic supplementary material:**

The online version of this article (10.1186/s12891-019-2523-2) contains supplementary material, which is available to authorized users.

## Background

Chronic low back pain (cLBP) affects more than 31 million Americans at any given time [1], has increased threefold in prevalence in a 10-year period [2], and results in $100–$200 billion per year in expenditures [3]. A large amount of these expenditures are attributed to 2 conditions associated with cLBP: intervertebral disc degeneration (IDD) and facet joint osteoarthritis (FOA) [4–10].

Several studies have suggested a link between cLBP and joint hypermobility, a condition in which joint range of motion is greater than normal [11, 12]. Joint hypermobility is more common in women than men [13] and occurs frequently in youth [14, 15]. It is associated with looseness of ligaments and the joint capsule more than excessive lengthening of the musculotendinous unit (i.e., flexibility), although an individual may present with both joint hypermobility and flexibility. Joint hypermobility may be advantageous for optimal performance of dancers, gymnasts, and musicians [16, 17], but individuals with joint hypermobility can experience considerable pain, disability, and decreased quality of life. Current treatment approaches include physical therapy, exercises and braces to stabilize joints, and self-management strategies for joint pain [14]. Since excessive joint mobility, in concert with insufficient muscular control, imposes altered stresses on joint tissues during functional activities [14, 18], joint hypermobility is a credible and potentially modifiable risk factor for low back pain and lumbar spine osteoarthritis (OA).

Only one prior cohort study has examined the association of joint hypermobility and lumbar spine disorders, but it was limited because only one participant had a Beighton Score ≥ 4/9 (general joint hypermobility) [19]. Furthermore, this study classified spine OA in only one way, as the presence of joint space narrowing, representing intervertebral disc space narrowing, and the presence of a vertebral osteophyte. While some have considered this definition of combined IDD and osteophytes to represent spine OA, others have defined OA of the spine to be isolated pathology of the facet joint, since there are known differences in their etiological processes of degeneration and therefore may have different associations with disease processes [5, 7]. We are unaware of previous studies that have examined the relationship between hypermobility and FOA in the lumbar spine. Research to add to and improve upon these prior limitations is needed to elucidate the relationship between joint hypermobility and spine OA and FOA. The purpose of this study was to examine the association of joint hypermobility and clinically relevant lumbar spine outcomes of pain, radiographic spine OA and FOA, and symptomatic spine OA or FOA in 3 large studies. We hypothesized that joint hypermobility would be associated with prevalent lumbar spine outcomes.

## Methods

### Data sources

The data for these analyses came from 3 different studies of participants with and without OA: the Generalized Osteoarthritis (GO) Study, the Genetics of Generalized Osteoarthritis (GOGO) Study, and the Johnston County Osteoarthritis (JoCo OA) Project. In each study, participants were sampled independently of their spine osteoarthritis, joint hypermobility and joint symptom status. Each study is described below and a table describing the period covered, inclusion/exclusion criteria, period at which Beighton Hypermobility Index was administered, characteristics of sample, period when outcome, exposures and covariates were measured is available in the Additional file 1.

#### Generalized osteoarthritis (GO) study

This case-control study examined the genetic differences of people with and without OA. Eligible participants were white, weighed < 300 pounds, and were unrelated by blood to any other participants. Beighton data were collected for all cases and controls. Participants were enrolled during 2002–2005 and were at least 45 years old and had hand OA with at least 3 enlarged finger joints (one must have been a distal interphalangeal joint, and each hand must have had at least one enlargement [same definition as GOGO below]). Participants could not have other types of arthritis (e.g., rheumatoid arthritis, lupus, gout, psoriatic arthritis), hemochromatosis, or ankylosing spondylitis. Controls were at least 60 years old and had no OA in hips, knees, or hands and did not have other types of arthritis, hemochromatosis, or ankylosing spondylitis.

#### Genetics of generalized osteoarthritis (GOGO) study

The purpose of the GOGO Study was to identify regions of the human genome among Caucasians that were associated with generalized (multi-joint) OA. The GOGO Study was a cohort study of 5 US sites (Durham, NC; Chapel Hill, NC; Baltimore, MD; Cleveland, OH; Chicago, IL) and 2 United Kingdom sites (Nottingham and Sheffield) [20]. Participants who were Caucasian were recruited during 2000–2002 from rheumatology clinics, hospital databases of OA patients, pre-existing OA cohorts, and the community using advertisements and word-of-mouth. A family was included in the study if at least two siblings met clinical GOGO hand OA criteria, defined as bony enlargement of ≥3 joints distributed across both hands, including bony enlargement of at least one DIP joint, and no more than three swollen metacarpophalangeal joints [20]. (Individuals with 3 involved joints on only one hand did not qualify). The total sample comprised 1145 families with 2728 participants. Radiography of the hands, hips, and knees (all sites) and lumbosacral spine (US sites) were completed. Self-reported past and measured present joint hypermobility data based on the Beighton scoring system were collected.

#### Johnston County osteoarthritis (JoCo OA) project

This ongoing, longitudinal study of OA includes African American (nearly 30% of the cohort) and white participants living in a largely rural county in North Carolina [21, 22]. Civilian, non-institutionalized residents aged 45+ years from 6 townships in Johnston County were enrolled between 1991 and 1998 (*n* = 3187) [23], and additional residents were enrolled from 2003 to 2004 (*n* = 1015). Participants completed follow-up clinic and interview data collection approximately every 5 years.

### Outcomes

Women of reproductive age (< 50 years old) were excluded from having lumbar spine radiographs in JoCo OA, and women of childbearing age completed a pregnancy test prior to radiography for the GO and GOGO studies. For participants eligible for radiography from all 3 studies, a lateral view of the lumbar spine was obtained using an identical protocol; lateral lumbar spine films were taken with the participant lying on his/her left side with the central beam centered at the lumbar spine. All lumbar spine radiographs for each study were graded at each lumbar level by a single musculoskeletal radiologist (JBR). The Burnett Atlas [24] was used to grade lumbar spine radiographic features of FOA, disc space narrowing (DSN), and osteophytes (OST). FOA was graded as absent or present at each lumbar level while DSN and OST were graded in a semi-quantitative fashion (0 = none, 1 = mild, 2 = moderate, and 3 = severe). The grading for OST was done for each superior and inferior aspect of the anterior face of the lumbar vertebra. In JoCo OA, we found moderate-to-strong intra-rater reliability for grading FOA with a kappa = 0.73, for DSN with a weighted kappa = 0.89, and for OST with a weighted kappa = 0.90 [25].

#### Radiographic spine OA and FOA

As in previous analyses [5, 7, 25, 26], a dichotomous variable for presence of lumbar spine OA at any lumbar spine level was derived, defined by the presence of both at least mild OST (either superior or inferior) and mild DSN at the same level of the lumbar spine. FOA was coded as 1 = present and 0 = absent at any level of the lumbar spine.

#### Low back pain

Symptoms were assessed for the low back with the following question: “On most days of any one month in the last year, did you have pain, aching, or stiffness in your low back?” Symptoms were considered present if the response was affirmative.

#### Symptomatic spine OA or FOA

Symptomatic spine OA or FOA was present if an individual had both low back pain and radiographic FOA or radiographic spine OA by the above definitions.

### Main exposures

Hypermobility in all 3 studies was defined by the Beighton criteria [27], the most widely used and reliable [28, 29] measure of hypermobility in the clinic and in research. The Beighton system was used to determine the participant’s ability to complete any of the following 9 maneuvers: (1) palms on the floor during forward trunk flexion with the knees extended, (2–3) right and left knee hyperextension of ≥10 degrees, (4–5) right and left elbow hyperextension of ≥10 degrees, (6–7) passive dorsiflexion of the right and left fifth fingers ≥90 degrees, and (8–9) passive apposition of the right and left thumbs to the forearm. One point is assigned for each completed maneuver, so the total Beighton score ranged from 0 (unable to perform any maneuver) to 9 (able to perform all maneuvers). In the research literature, individuals with a score of at least 4 are frequently classified as having general joint hypermobility [30–35], although other cutoff values [28, 36] and use of a continuous score have been considered [27, 37]. The primary definition used in the present analyses is a cutoff of 4, where a score of 4–9 is defined as the presence of general joint hypermobility [30–35, 37]. We also analyzed the trunk flexion maneuver (question #1 of the Beighton system described above) as a separate individual exposure due to its specific functionality with the lumbar spine and low back pain.

### Covariates

Potential confounders for within-study analyses included race (African American [JoCo OA only] or white), and within- and between-study confounders included sex, age (years), body mass index (BMI, kg/m^2^), and history of any back injury (yes/no) by questionnaire. Height in centimeters and weight in kilograms were measured in each study (with a stadiometer and balance beam scale) and used to calculate BMI.

### Statistical analysis

**Fig. 1:**
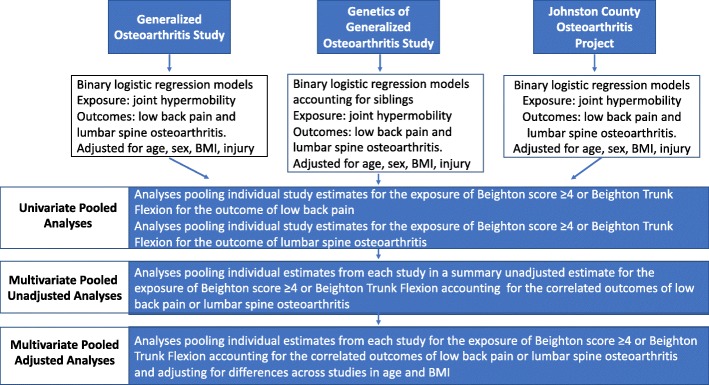
Illustration of the process from primary analysis for each study, univariate pooled analysis, multivariate unadjusted pooled analysis and multivariate pooled and adjusted analysis performed

Participants with complete data for the outcomes, exposures, and covariates from a single study visit were included in these cross-sectional analyses. We conducted descriptive analyses for covariates and outcome variables of each study in the form of means and standard deviations or counts and percentages, as appropriate. Analyses of individual studies were conducted while adjusting for available covariates for each outcome with binary logistic regression. In the GOGO study only, logistic analysis using generalized estimating equations were used to cluster by family structure (i.e., siblings). We then combined individual study estimates (univariate models) into 2 pooled analyses, one for Beighton score ≥ 4 and one for the individual Beighton trunk flexion maneuver combining one parameter over several outcomes computed with DerSimonian and Laird random-effects models [38] with inverse variance weighting. Pooled estimates (pooled odds ratios [pORs]) were generated for each exposure by combining estimates of several related outcomes (multivariate models). These models were estimated with a methods-of-moments procedure, which is a multivariate generalization of the DerSimonian and Laird random effects model [39]. Model within-study correlations were set at *r* = 0.7 to match the findings from our covariance matrices, and an unstructured between-study variance covariance matrix was specified. We then entered a single covariate for each of these models (multivariate adjusted models) to determine the effect of between-study variations in demographics or clinical characteristics. To maintain model parsimony, covariates with model *p*-values < 0.05 were retained for the final model. Figure 1 illustrates the analysis process from primary analysis of each individual study, univariate pooled analysis, multivariate pooled analysis and multivariate pooled and adjusted analysis. We then calculated a change-in-estimate (difference in unadjusted multivariate beta coefficient minus the absolute value in the adjusted multivariate beta coefficient) to determine the relative amount of adjustment. An I^2^ statistic was calculated from the multivariate models for each outcome [40]. All analyses were conducted in Stata v.15 (Stata Corp., College Station, TX).

## Results

**Fig. 2:**
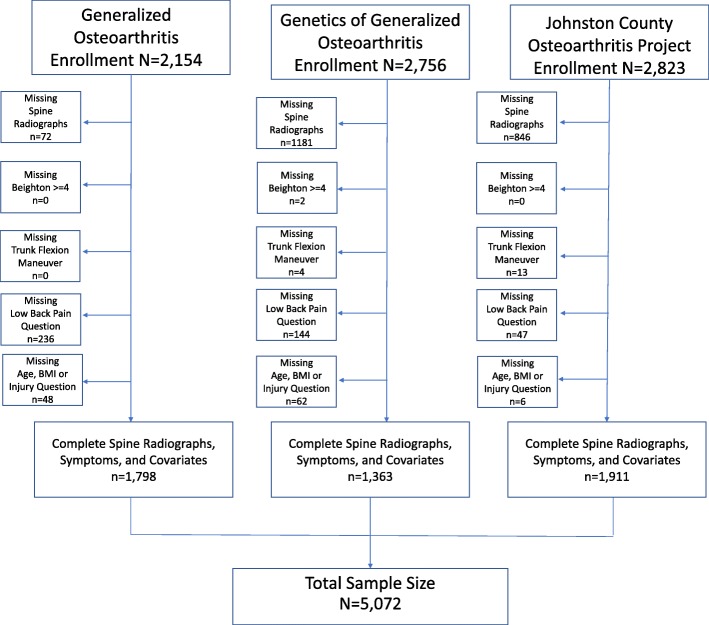
Sample sizes and missing data for each study included in the pooled analysis

Figure 2 illustrates the enrollment of participants into each study and missing data for exposures, outcomes, and covariates. Exclusion of women of childbearing age was the primary reason for missing lumbar spine radiographs in JoCo OA. Regulatory restrictions preventing pelvic radiographs at some sites in this multi-site study were primarily responsible for missing lumbar spine radiographs from the GOGO study. The total sample size for pooled analyses was *n* = 5072.

Table 1 describes the participant and clinical characteristics across the 3 studies. Each study contributed a similar sample size. Similarities were found for age, with a slightly higher mean age for the GO study. Women made up the majority of each study, with JoCo OA having a slightly lower proportion. JoCo OA had the only biracial cohort recruiting African Americans (32.4%). The GO study had a lower mean BMI compared with GOGO and JoCo OA (27.5% vs. 29.1 and 30.9%, respectively). Back injury was similarly rare (~ 2%) across all 3 studies. Low back pain occurred more frequently among the GOGO cohort (65.1%) than in GO (37.9%) and JoCo OA (40.1%). A Beighton score ≥ 4 was substantially more common in the GO study (11.0%) than either the GOGO (4.2%) or JoCo OA (6.4%) studies. Similarly, the proportion of participants able to perform the trunk flexion maneuver was higher in GO (12.9%) than in GOGO (6.9%) or JoCo OA (5.4%).

**Table 1:** Participant characteristics and disease statuses of those included from the 3 studies

	Generalized OA n (%) or mean (SD) *n* = 1798	Genetics of Generalized OA n (%) or mean (SD) *n* = 1363	Johnston County OA Project n (%) or mean (SD) *n* = 1911
Age, yrs	69.3 (8.4)	66.0 (10.0)	66.5 (10.1)
Women	1290 (71.8%)	1068 (78.4%)	1233 (64.5%)
African American	0 (0%)	0 (0%)	620 (32.4%)
Body mass index, kg/m^2^	27.5 (5.1)	29.1 (6.3)	30.9 (6.5)
Back injury	38 (2.1%)	34 (2.5%)	45 (2.4%)
Low back pain	681 (37.9%)	887 (65.1%)	767 (40.1%)
Beighton ≥4	197 (11.0%)	57 (4.2%)	122 (6.4%)
Trunk flexion maneuver	231 (12.9%)	94 (6.9%)	103 (5.4%)
Spine OA	1039 (57.8%)	791 (58.0%)	1136 (59.5%)
Facet OA	1333 (74.1%)	903 (66.3%)	1305 (68.3%)
Symptomatic spine OA	446 (24.8%)	556 (40.8%)	474 (24.8%)
Symptomatic facet OA	517 (28.8%)	598 (43.9%)	521 (27.3%)

Facet OA = presence of facet joint OA at any lumbar spine level; OA = osteoarthritis; Spine OA = combination of at least mild disc space narrowing and presence of a mild osteophyte at the same lumbar level; SD = standard deviation

**Fig. 3:**
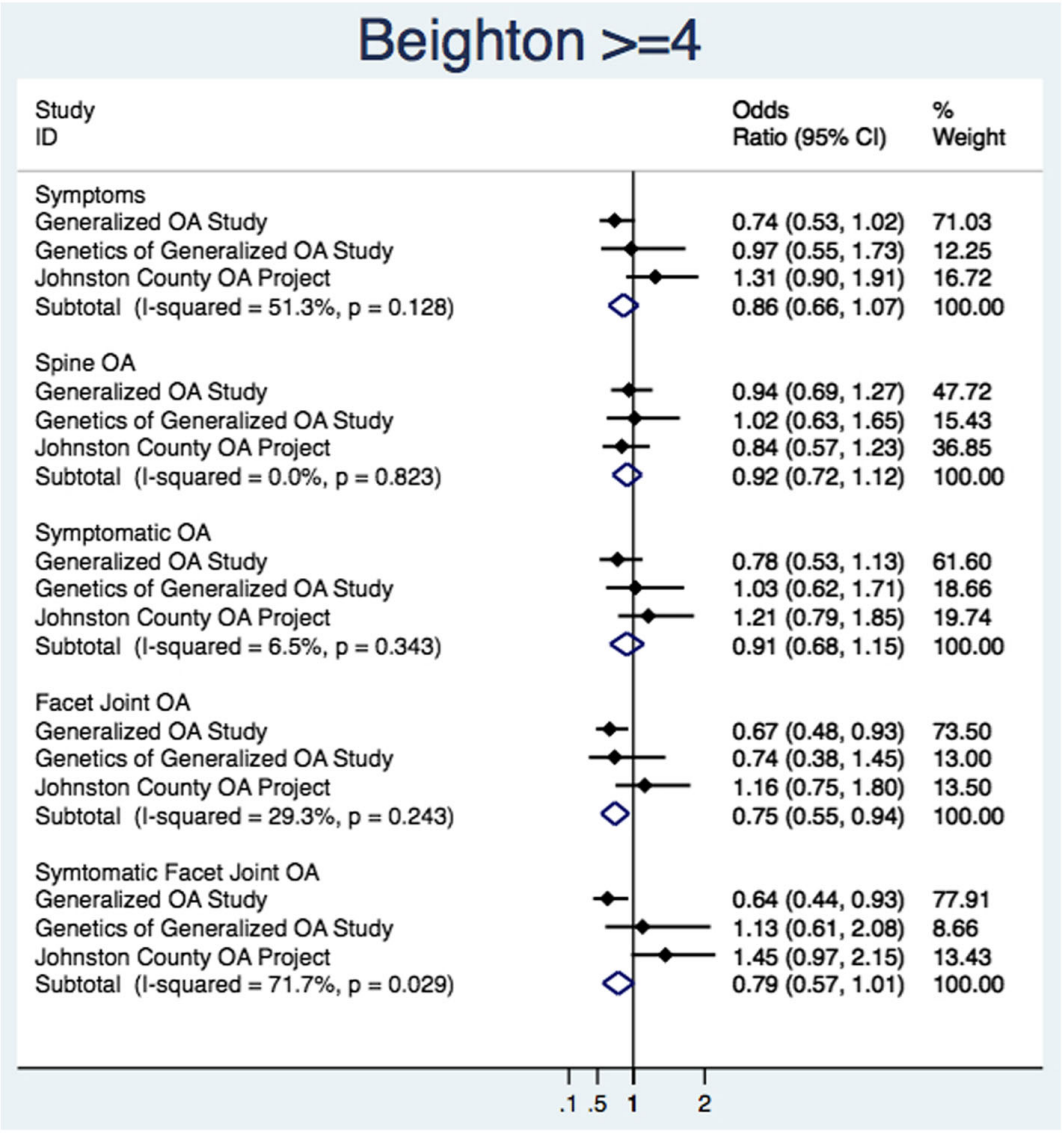
Primary estimate and univariate pooled estimates with the primary exposure of Beighton score ≥ 4 and the outcomes of low back symptoms, spine osteoarthritis, and facet joint osteoarthritis

Figure 3 illustrates the individual study estimates and pooled estimates for each outcome from univariate random effect models for the exposure of Beighton total score ≥ 4. Individual estimates were generally inconsistent between studies, with I^2^ values ranging from 0.0 to 71.7%. The only significant pooled estimate was for FOA. Among those with a Beighton ≥4 score, there was a 25% reduction in the odds of FOA (pOR = 0.75 [95% CI 0.55, 0.94]).

**Fig. 4:**
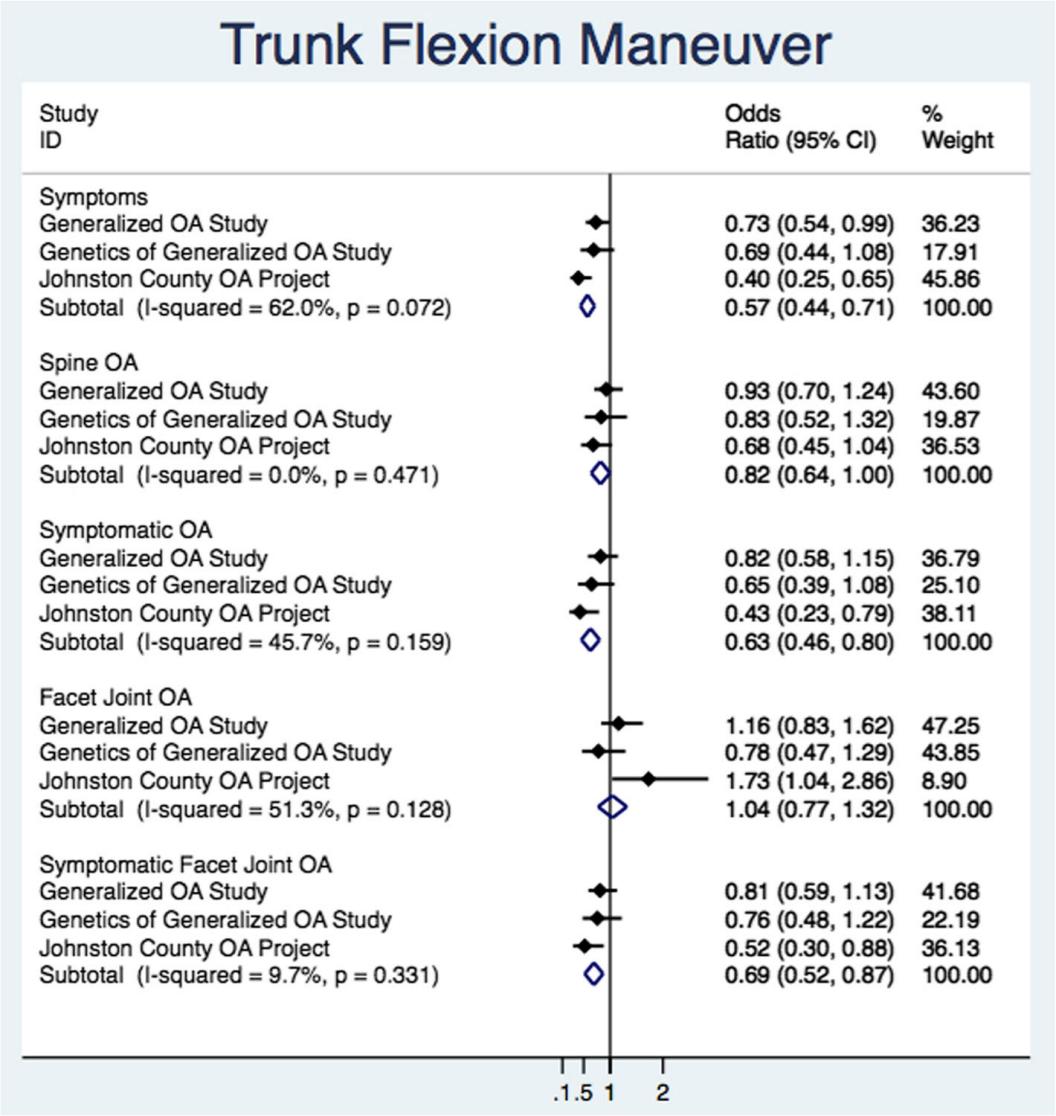
Primary estimate and univariate pooled estimates with the primary exposure of trunk flexion maneuver and spine osteoarthritis

Figure 4 illustrates the individual study estimates and pooled estimates for each outcome from univariate random effect models for the trunk flexion maneuver. Individual study estimates were generally consistent on the same side of the null. Between-study variation (I^2^) ranged from 0.0 to 62.0%. Presence of low back pain had the largest influence on the strength of association. Pooled estimates varied at pOR = 0.57 (95% CI 0.44, 0.71) for the presence of low back pain, pOR = 0.63 (95% CI 0.46, 0.80) for symptomatic spine OA, and pOR = 0.69 (95% CI 0.52, 0.87) for symptomatic FOA.

**Fig. 5:**
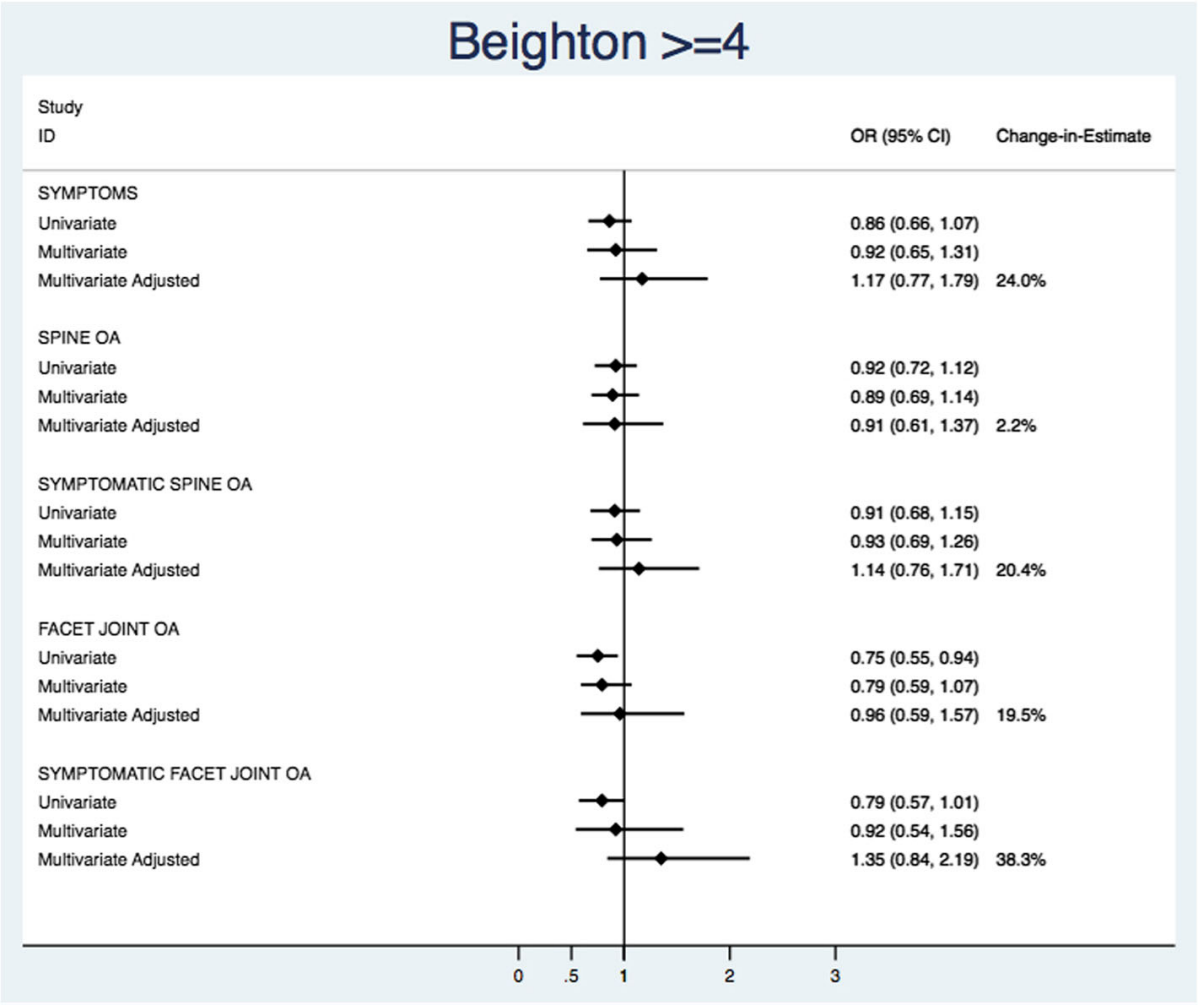
Summary of univariate, multivariate, multivariate adjusted pooled estimates, and change in estimate from multivariate and multivariate adjusted pooled estimates for the Beighton≥4

**Fig. 6:**
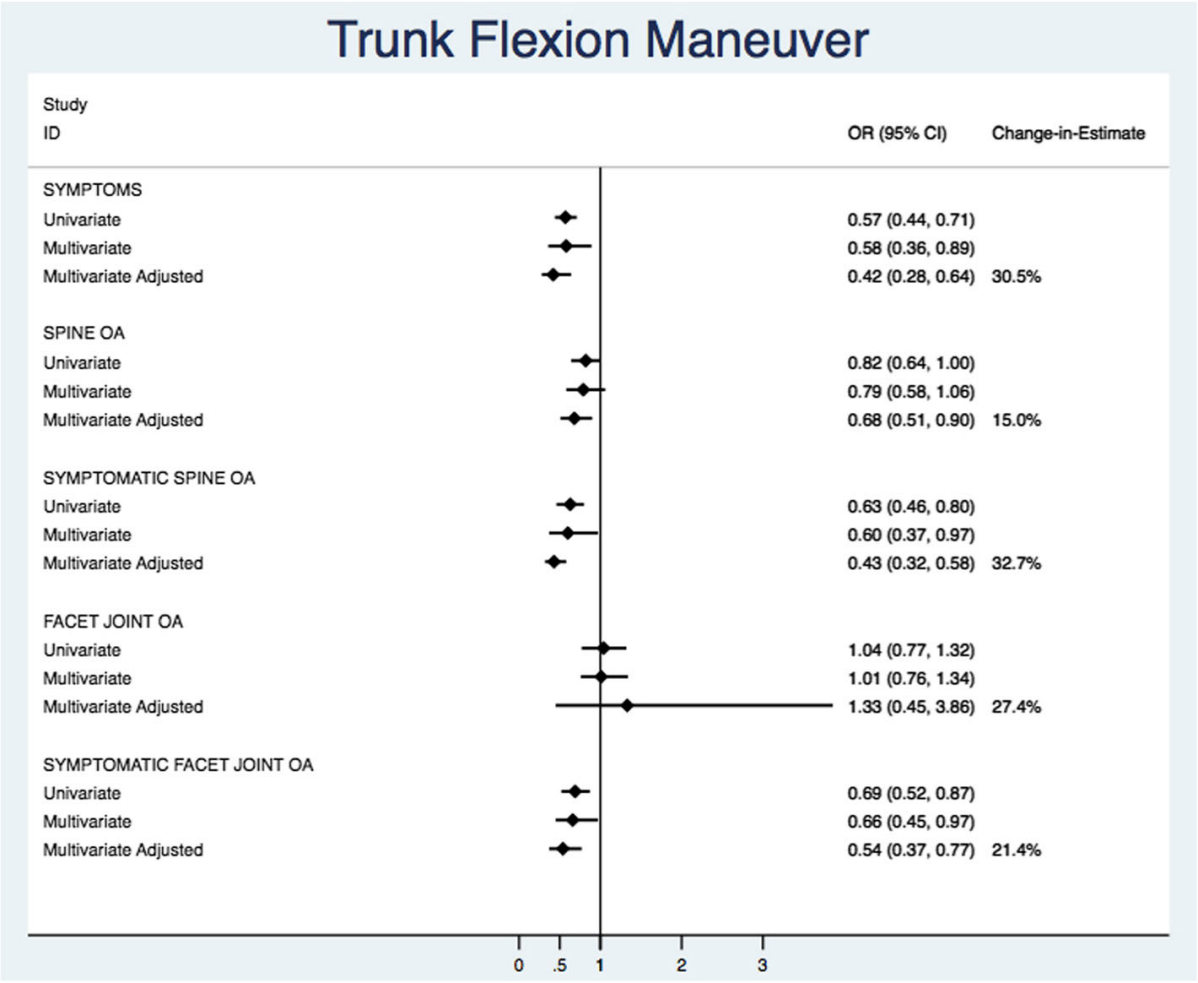
Summary of univariate, multivariate, multivariate adjusted pooled estimates, and change in estimate from multivariate and multivariate adjusted pooled estimates for the trunk flexion maneuver

Figure 5 illustrates the univariate, multivariate, multivariate adjusted models, and change-in-estimate for Beighton score ≥ 4. Univariate estimates had wider confidence intervals and were closer to the null when compared with multivariate and multivariate adjusted estimates. Despite large changes between the unadjusted and adjusted (age and BMI) model estimates, no multivariate or multivariate adjusted parameter estimates were significantly associated with any of the spine outcomes. For the trunk flexion maneuver (Fig. 6), univariate estimates were closer to the null but confidence intervals were relatively similar in width. When adjusting for between-study variations in mean BMI, the ability to conduct the trunk flexion maneuver was significantly and strongly associated with the decreased odds of low back pain (pOR = 0.42 [95% CI 0.28, 0.64]; I^2^ = 30.5%), spine OA (pOR = 0.68 [0.51, 0.90]; I^2=^15.0%), symptomatic spine OA (pOR = 0.43 [0.32, 0.58]; I^2^ = 32.7%), and symptomatic FOA (pOR = 0.54 [0.37, 0.77]; I^2^ = 21.4%). Facet joint OA was the only estimate on the opposite side of the null with a large degree of imprecision (pOR = 1.33 [0.45, 3.86]; I^2^ = 27.4%). Changes in estimates (15.0–32.7%) were large between unadjusted and adjusted multivariate models. In the multivariate models, the between-study variation (I^2^) ranged from 6.0 to 44.1% for Beighton ≥4 and 22.0 to 90.0% for trunk flexion manuever (Additional file 2).

## Discussion

We set out to combine the estimates from 3 separate studies for 2 different categories of an uncommon exposure (< 13%) with joint hypermobility in these studies of older adults) and several correlated lumbar spine outcomes. These 3 unique data sources have identical measurements of the exposure, outcomes, and covariates, allowing for comparing and combining of results of the joint hypermobility-lumbar spine outcomes by individually estimating multivariate and multivariate adjusted effects within and across data sources. By doing so, we were able to descriptively and qualitatively describe the differences in effect estimates and the overall pooled effects of joint hypermobility and outcomes of lumbar spine OA and FOA. We found that those with a positive trunk flexion maneuver were significantly less likely to have low back pain even in the presence of radiographic spine OA or FOA.

Our study found that participants with the ability to complete the trunk flexion maneuver had significantly lower odds of spine degeneration in the form of DSN and FOA. There are consistent associations between low back pain and these lumbar structures, suggesting that low back pain may play a pivotol role in the ability to perform trunk flexion. Previous studies on hypermobility and the lumbar spine are scarce; there are only 2, and both recruited only adolescents and young males [11, 12]. In these studies, young males with joint hypermobility (*n* = 32) had higher intervertebral disc heights (less DSN) compared with age-matched controls (*n* = 32) [11] and, although not statistically significant, among adolescents, had a positive association between low back pain and joint hypermobility [12]. The mechanistic reasons for the protective effect we identified in our study are unclear and may be related to biomechanical differences in lumbar segment motion or biological differences in the proteoglycan content of the intervertebral disc between those with and without joint hypermobility. This study did not find that total score on the Beighton scale, representing generalized hypermobility, was significantly associated with low back pain or lumbar spine degeneration. One reason for this lack of association may be that only one factor of the Beighton scale is related to lumbar spine function.

We combined results from separate studies with correlated outcomes while adjusting for differences between studies using clinical and demographic characteristics. The two degenerative structures we assessed in the lumbar spine, IDD and FOA, have been known to have some correlation with low back pain and are strongly correlated with one another [7]. This correlation is likely the reason for the wider confidence intervals in the estimates provided by the multivariate models when compared with the univariate analysis. In multivariate adjusted models, we found that between-study variations in mean BMI and to some extent mean age have substantial influences on the parameter estimates. While this method provided an efficient approach to account for correlated outcomes and between-study variations in measured covariates, this study is not without limitations. All of our analyses are cross-sectional; therefore, we are unable to determine a causal relationship. The results of this study do not suggest any changes in current approaches for managing individuals with joint hypermobility. Although all three studies used identical measures and assessment of radiographic features, the sampling differences and designs (cohort and case-control) that differ between the studies may be one reason for the heterogeneity found in some of our estimates. The cross-sectional nature and age of recruitment also limits our ability to assess the extent to which misclassification of the exposure (i.e., participants with hypermobility in adolesence who develop joint stiffening as they age being classified as “not hypermobile” at the time of study assessment) may influence our exposure estimates. We were able to adjust for potential confounding covariates from each of our studies for the primary analysis. However, we were not able to adjust for all possible factors and therefore cannot rule out residual confounding. In addition, although the proportion of spine OA and FOA appears to be similar across studies suggesting consistency in factors that may influence the occurrence of spine degeneration, we did find differences across studies for demographic and clinical characteristics. Although we were able to account for these demographic and clinical characteristic differences across studies in our models, we were unable to account for all potential confounders that may influence the occurrence of these outcomes across studies, such as, but not limited to, pain medication use, occupational characteristics, physical activity and race.

## Conclusion

Compared with participants who cannot complete the trunk flexion maneuver, those who can are less likely to have low back pain or symptomatic spine degeneration. Flexibility of the spine and surrounding musculature could be explored in future studies to determine its role in avoiding or managing these conditions. The approach taken here for this work may have implications for those who may be working with uncommon exposures and correlated outcomes from separate data sources. Longitudinal studies are needed and should include younger participants to determine the causal relationship between joint hypermobility and spine degeneration and low back pain.

## Additional files

Additional file 1: Multivariate and adjusted multivariate models for the 2 categories of hypermobility across study outcomes. This table provides the pooled estimates, I^2^ values and change-in-estimate, from the multivariate and adjusted multivariate models for the relationship of the two expsoures of Beighton ≥4 and trunk flexion maneuver and outcomes of low back symptoms, spine osteoarthritis (OA), symptomatic spine OA, facet joint OA and symptomatic facet joint OA. (DOCX 14 kb)

Additional file 2: Period covered, inclusion/exclusion criteria, period at which Beighton Index data were collected, when the outcome, exposure and covariates where measured. This table provides the period covered, inclusion/exclusion criteria, Beighton Data Collection Time Period, percentage of participants with spine osteoarthritis (OA), percentage of participants with moderate spine OA, percentage of participants with facet joint OA, the time period for which spine OA was measured, and the time period for which low back pain was measured in each of the three cohorts. (DOCX 17 kb)

## Abbreviations

BMI: Body mass index
CI: Confidence interval
cLBP: Chronic low back pain
DSN: Disc space narrowing
FOA: Facet joint osteoarthritis
GO Study: Generalized Osteoarthritis Study
GOGO Study: Genetics of Generalized Osteoarthritis Study
IDD: Intervertebral disc degeneration
JoCo OA Project: Johnston County Osteoarthritis Project
OA: Osteoarthritis
OST: Osteophyte
pOR: Pooled odds ratio
