# Supplementary material for: Relationship of joint hypermobility with low Back pain and lumbar spine osteoarthritis
Source: BMC Musculoskelet Disord. 2019 Apr 9;20:158. doi: 10.1186/s12891-019-2523-2 (PMC6456963; doi:10.1186/s12891-019-2523-2)
Supplement: Supplementary file 1 — Multivariate and adjusted multivariate models for the 2 categories of hypermobility across study outcomes. This table provides the pooled estimates, I2 values and change-in-estimate, from the multivariate and adjusted multivariate models for the relationship of the two expsoures of Beighton ≥4 and trunk flexion maneuver and outcomes of low back symptoms, spine osteoarthritis (OA), symptomatic spine OA, facet joint OA and symptomatic facet joint OA. (DOCX 14 kb) [file 12891_2019_2523_MOESM1_ESM.docx]

**Additional File 1.** Multivariate and adjusted multivariate models for the 2 categories of hypermobility across study outcomes.

| Exposure | Outcome | Multivariate pooled estimate (95% CI) | I^2^ | Multivariate adjusted pooled estimate (95% CI) | Change in estimate |
| --- | --- | --- | --- | --- | --- |
| Beighton ≥4 |  |  |  |  |  |
|  | Symptoms | 0.92 (0.65, 1.31) | 64.0% | 1.17 (0.77, 1.79) | 24.0% |
|  | Spine OA | 0.89 (0.69, 1.14) | 0.0% | 0.91 (0.61, 1.37) | 2.2% |
|  | Symptomatic spine OA | 0.93 (0.69, 1.26) | 10.0% | 1.14 (0.76, 1.71) | 20.4% |
|  | Facet joint OA | 0.79 (0.59, 1.07) | 51.0% | 1.14 (0.76, 1.57) | 19.5% |
|  | Symptomatic facet joint OA | 0.92 (0.54, 1.56) | 74.0% | 1.35 (0.84, 2.19) | 38.3% |
| Trunk flexion maneuver |  |  |  |  |  |
|  | Symptoms | 0.58 (0.36, 0.89) | 90.0% | 0.42 (0.28, 0.64) | 30.5% |
|  | Spine OA | 0.79 (0.58, 1.06) | 22.0% | 0.68 (0.51, 0.90) | 15.0% |
|  | Symptomatic spine OA | 0.60 (0.37, 0.97) | 87.0% | 0.43 (0.32, 0.58) | 32.7% |
|  | Facet joint OA | 1.01 (0.76, 1.34) | 30.0% | 1.33 (0.45, 3.86) | 27.4% |
|  | Symptomatic facet joint OA | 0.66 (0.45, 0.97) | 63.0% | 0.54 (0.37, 0.77) | 21.4% |

Beighton ≥4 multivariate adjusted estimates for age and body mass index (BMI); trunk flexion maneuver adjusted for BMI only. CI=confidence interval; OA=osteoarthritis.
