# Supplementary material for: Relationship of joint hypermobility with low Back pain and lumbar spine osteoarthritis
Source: BMC Musculoskelet Disord. 2019 Apr 9;20:158. doi: 10.1186/s12891-019-2523-2 (PMC6456963; doi:10.1186/s12891-019-2523-2)
Supplement: Supplementary file 2 — Period covered, inclusion/exclusion criteria, period at which Beighton Index data were collected, when the outcome, exposure and covariates where measured. This table provides the period covered, inclusion/exclusion criteria, Beighton Data Collection Time Period, percentage of participants with spine osteoarthritis (OA), percentage of participants with moderate spine OA, percentage of participants with facet joint OA, the time period for which spine OA was measured, and the time period for which low back pain was measured in each of the three cohorts. (DOCX 17 kb) [file 12891_2019_2523_MOESM2_ESM.docx]

Additional File 2. Period covered, inclusion/exclusion criteria, period at which Beighton Index data were collected, when the outcome, exposure and covariates where measured.

| Characteristics | Generalized Osteoarthritis  (n=2,154) | Genetics of Generalized Osteoarthritis (n=2,756) | Johnston County Osteoarthritis Project (n=2,823) |
| --- | --- | --- | --- |
| Period Covered | 2002-2005 | 2000-2002 | 2003-2010 |
| Inclusion / Exclusion Criteria | Case-control study. Eligible participants were Caucasian, weighed less than 300 pounds, and were unrelated by blood to any other participants. Cases were at least 45 years old and had hand OA based on the same definition as GOGO in next column. They could not have other types of arthritis (e.g., rheumatoid arthritis, lupus, gout, psoriatic arthritis), hemochromatosis, or ankylosing spondylitis. Controls were at least 60 years old without OA (in hips, knees or hands), other types of arthritis, hemochromatosis, or ankylosing spondylitis. | Participants were included if they were Caucasian, fulfilled clinical GOGO hand OA criteria (bony enlargement of > 3 hand joints distributed bilaterally, including bony enlargement of > 1 distal interphalangeal joint and no more than 3 swollen metacarpophalangeal joints), and had > 1 sibling who agreed to participate in the study. | Civilian, non-institutionalized residents aged 45+ years from six townships in Johnston County who were African American or Caucasian men and women. |
| Beighton Data Collection Time Period | 2002-2005 | 2000-2002 | 2003-2010 |
| Spine Osteoarthritis, (%) | 57.8% | 58.0% | 59.5% |
| Moderate Spine Osteoarthritis, (%) | 21.8% | 21.7% | 25.5% |
| Facet Osteoarthritis, (%) | 74.1% | 66.3% | 68.3% |
| Spine Osteoarthritis Time Period | 2002-2005 | 2000-2002 | 2003-2010 |
| Low Back Pain Time Period | 2002-2005 | 2000-2002 | 2003-2010 |
